# Hypertension and Reproduction

**DOI:** 10.1007/s11906-020-01036-2

**Published:** 2020-03-13

**Authors:** Peter M. Nilsson, Margus Viigimaa, Aleksander Giwercman, Renata Cifkova

**Affiliations:** 1 Department of Clinical Sciences, Lund University, Lund, Sweden; 2 Skåne University Hospital, Malmö, Sweden; 3 Heart Health Centre of North Estonia Medical Centre, and Centre for Cardiovascular Medicine, Tallinn University of Technology, Tallinn, Estonia; 4 Department of Translational Medicine, Skåne University Hospital, Lund University, Malmö, Sweden; 5 Center for Cardiovascular Prevention, First Faculty of Medicine and Thomayer Hospital, Charles University in Prague, Prague, Czech Republic; 6 Department of Medicine II, First Faculty of Medicine, Charles University in Prague, Prague, Czech Republic

**Keywords:** Assisted reproductive technologies, Erectile dysfunction, Hypertensive disorders in pregnancy, Low birth weight, Male hypogonadism, Oral contraception

## Abstract

**Purpose of Review:**

Many aspects of reproduction have been associated with increased blood pressure and impaired glucose metabolism that reveals a subsequent increased risk of cardiovascular disease. The aim of this review is to assess reproductive life factors associated with an increased risk of hypertension and cardiovascular disease, e.g., early life programming, sexual, and reproductive health in men and women.

**Recent Findings:**

Impaired fetal growth, with low birth weight adjusted for gestational age, has been found associated with hypertension in adulthood. Erectile dysfunction, currently considered an early diagnostic marker of cardiovascular disease preceding the manifestation of coronary artery disease by several years, frequently coexisting with hypertension, could also be exacerbated by some antihypertensive drugs. Male hypogonadism or subfertility are associated with increased cardiovascular risk. Hypertensive disorders in pregnancy including preeclampsia represent a major cause of maternal, fetal and neonatal morbidity, and mortality. The risk of developing preeclampsia can be substantially reduced in women at its high or moderate risk with a low dose of acetylsalicylic acid initiated from 12 weeks of gestation. An increased risk of hypertension in women following invasive-assisted reproductive technologies has been newly observed. Blood pressure elevation has been noticed following contraceptive pill use, around the menopause and in postmenopausal age. Furthermore, drug treatment of hypertension has to be considered as a factor with a potential impact on reproduction (e.g., due to teratogenic drug effects).

**Summary:**

In summary, a deeper understanding of reproductive life effects on hypertension and metabolic abnormalities may improve prediction of future cardiovascular disease.

## Introduction

The reproductive system is shaped by evolution and is of fundamental importance for survival of mankind. Many complicated processes interact to control reproductive capacity and fertility [1, 2]. The reproductive system is sensitive and vulnerable with potential consequences of infertility, fetal loss, pregnancy-related diseases, and hormonal imbalance to impaired survival of new-born children. The World Health Organization (WHO) has, traditionally, focused on reproductive health and preventive measures concerning mother and child as an investment into future adult health [3]. Recently, this issue has expanded to include preconception counseling to improve the health of young women planning for pregnancy, (e.g., folic acid supplementation) [4].

In cardiovascular medicine, recent guidelines have emphasized the role of reproductive history in women when evaluating cardiovascular risk [5], i.e., history of pregnancy-induced complications (gestational hypertension, preeclampsia [6], gestational diabetes), premature menopause, or exposure to sex hormones such as hormonal contraceptives or hormonal replacement therapy in menopause that may increase blood pressure (BP) levels. Similarly, in men, information on sexual and reproductive history, as exemplified by the role of erectile dysfunction (ED) as an early marker of endothelial dysfunction and atherosclerosis [7] should be collected. Further, information on cardiometabolic comorbidities that have been recently linked to male infertility should also be obtained [8]. A list of reproductive factors associated with hypertension is provided in Table 1.

**Table 1:** (A) Reproductive factors associated with hypertension; versus (B) hypertension, or its treatment, associated with reproductive factors

(A) Reproductive factors associated with hypertension; versus Pregnancy complications Fetal growth retardation Low birth weight Contraceptives Hormonal replacement therapy Hypogonadism, infertility	
(B) Hypertension, or its treatment, associated with reproductive factors Preconception (pre-existing) hypertension Antihypertensive drugs and erectile dysfunction Teratogenic effects by RAS blockers	

*RAS* renin-angiotensin-system

The aim of this review is to explore examples of factors of reproductive life, including early life programming, sexual health, male hypogonadism, and female reproductive history that have been associated with increased risk of hypertension and cardiovascular disease (CVD) in later life. Research devoted to a better understanding of these observed associations could provide new insights and help develop new strategies for the prevention and treatment of hypertension and CVD, e.g., targeting new risk groups for screening and intervention. Health problems linked to reproduction could increase the risk of hypertension, while hypertension or its drug treatment may have an impact on reproductive risk conditions, e.g., preeclampsia and erectile dysfunction.

### Early Life Programming of Adult Hypertension and Cardiovascular Risk

Several studies have documented low birth weight, or low birth weight in relation to gestational age (small-for gestational-age; SGA), a possible consequence of intra-uterine growth retardation (IUGR), as risk markers for future BP elevation and hypertension in adult life. However, these exposures have not always been measured in the same study. Early studies by Gennser [9], Barker [10], and Lithell [11] have shown evidence to support this association, first in young males [9, 12], and later in adult men and women. Several meta-analyses have confirmed the relationship [13, 14], even though not always consistent as some studies failed to show the association [15]. This inconsistency likely reflects methodological differences among studies, i.e., the imprecise determination of gestational age and screening measurement of (office) BP. The association between adverse early life factors and adult BP elevation has been confirmed in diverse ethnic groups in various parts of the world [16, 17].

Low birthweight/SGA were also found to be associated with an increased risk for the development of ischemic heart disease [18] and type 2 diabetes in adulthood [19]. Ischemic heart disease is related to both neonatal and postneonatal morbidity and, therefore, to both intrauterine and postnatal environments [18]. The links may include elevated BP and as yet unknown processes established in early postnatal life. According to Barker [10], this could be a consequence of poor nutrition in pregnant women, the effects of unhealthy lifestyle (smoking, alcohol consumption) or impaired placental function. Other researchers have shown that the immediate postnatal period is also important. Children born small-for-gestational age (SGA) because of IUGR are preprogrammed in utero to enter a world of calorie restriction, but if they are exposed to a postnatal world of nutritional overflow (“mis-match”) with rapid catch-up growth, their risk for developing CVD could increase further in adulthood, e.g., present as adverse cardiometabolic health [20]. This hypothesis is further developed in the Developmental Origin of Health and Disease (DOHaD) concept, as promoted by the DOHaD Society (www.dohad.org). Postnatal feeding may be of great importance for the different unfavorable postnatal phenotypes, and breast feeding is strongly recommended to prevent them.

#### Mechanisms Linking Early Life Factors with Cardiovascular Risk

Some studies have documented that children born with SGA or with a low birth weight have disturbed neuroendocrine function with a relative adrenal hyperactivity in adulthood [21]. Increased sympathetic nervous activation has been also shown in animal studies [22]. In addition, impaired fetal growth has been associated with a lower number of nephrons [23] and thus a tendency for impaired renal function, causing impaired sodium excretion. Finally, impaired insulin sensitivity and thereby increased insulin resistance due to the defects in the evolution of skeletal muscle tissue and its metabolism [24] could contribute to the development of hyperinsulinemia and, consequently, increased sodium retention, inducing BP elevation. An association between birth weight and insulin resistance was found, but the association was reversed for those born preterm. The authors emphasized the need to “stratify by gestational age” in analysis of both previous and future studies [24].

The possible link between early life factors and increased cardiovascular risk in the offspring of pregnant women with poor nutrition, unhealthy lifestyle (smoking, alcohol intake) or impaired placental function has remained controversial. Some researchers argue that genetic factors may explain the link between hypertension in the mother, SGA of the newborn, and development of hypertension in the offspring in adult life [25].

Changes in the vascular system of growth-retarded babies include less developed capillaries and probably lower elastin accumulation in the media of elastic arteries, with a possible consequence of impaired elasticity promoting arterial stiffness [26] and increased augmentation pressure index [27]. The divergent results from various studies could be explained by differences in measurement techniques, as well as by the differential effects of impaired fetal growth on the one hand, and those of prematurity on the other. Both conditions could lead to low birth weight by different mechanisms.

### Sexual Life, Erectile Dysfunction, and Cardiovascular Risk

Sexual function represents an integral part of human general health and wellbeing. Erectile dysfunction (ED), a condition frequently co-existing with hypertension, contributes significantly to the impaired health–related quality of life of both hypertensive patients and their sexual partners [28]. The prevalence of ED in hypertensive individuals is approximately twice that of normotensive individuals. However, ED remains underreported, underrecognized, and undertreated in hypertensive patients [29]. ED was included for the first time ever in the 2013 ESH/ESC Guidelines for the management of arterial hypertension [30].

Erectile dysfunction is considered to be of vasculogenic origin in the vast majority of patients. It is an early diagnostic marker of CVD, preceding the manifestation of coronary artery disease by 3–5 years [31]. Consistent with the “artery size hypothesis”, it has been assumed that vascular lesions predisposing to CVD develop earlier in the small diameter penile arteries (1–2 mm) than in larger arteries, including the coronary arteries (3–4 mm) [32].

Further, ED shares modifiable risk factors with hypertension. Randomized clinical trials have shown lifestyle modification to be of clinical benefit in improving ED [33]. Lifestyle measures that reduce the risk of ED, hypertension, and the risk of BP-related cardiovascular complications include smoking cessation, weight reduction and maintenance, regular physical exercise, moderation of alcohol consumption, and dietary changes.

#### Effects of Antihypertensive Drugs on Erectile Function

Data regarding the effects of antihypertensive drug therapy on ED are limited, usually address monotherapy only, and come mainly from observational studies. Data from randomized studies addressing combination therapy are urgently needed [34, 35]. To date, ED has never been defined as a primary endpoint in any large clinical trial of antihypertensive drugs.

Available data suggest that older classes of antihypertensive agents (thiazides, beta-receptor blockers) are inferior to the newer ones (RAS blockers, calcium antagonists) regarding ED [36]. In particular, the adverse effects of beta-blockers have been repeatedly confirmed, with the only possible exception of nebivolol, which has been reported to exert beneficial vasodilatory effects, possibly due to nitric oxide (NO) modulation.

Diuretics are also believed to impair ED, even when used in combined antihypertensive therapy [35]. The type and dosage of the diuretic may be of importance. Although data regarding calcium antagonists and angiotensin-converting enzyme (ACE) inhibitors are not yet definitive, a neutral effect on ED has been reported [36]. Angiotensin receptor blockers (ARBs) may positively affect ED [37] and have been recommended as first-line treatment in patients with pre-existing ED. However, the ONTARGET/TRANSCEND trials did not demonstrate a benefit of an ARB on ED when added to previous multidrug regimen in high-risk hypertensive patients [38]. Available data suggest significant benefits in erectile function when prior antihypertensive drug therapy is switched to either nebivolol or a renin-angiotensin system blocker.

#### Phosphodiesterase-5 Inhibitors in Patients with Hypertension

Over the past 15–20 years, phosphodiesterase-5 (PDE-5) inhibitors have emerged as an efficacious therapy for ED. The use of PDE-5 inhibitors in hypertensive patients provides important benefits. PDE-5 inhibitors exert their beneficial vasodilating effects, i.e., contributing to improved erectile function, mainly through improved adherence. Hypertensive men with ED are more likely to initiate and add antihypertensive medication when receiving PDE-5 inhibitors [39].

PDE-5 inhibitors are, in general, well tolerated and relatively safe, but their use should follow appropriate consultation. In hypertensive patients treated with nitrates, PDE-5 inhibitors decrease systolic and diastolic BP. When PDE-5 inhibitors are co-administered with organic nitrates such as nitroglycerine and isosorbide mononitrate, unpredictable vasodilatory, and hypotensive effects may be observed. Co-administration of PDE-5 inhibitors and nitrates is therefore contraindicated.

Finally, ED is included in both European and US hypertension and cardiovascular prevention guidelines [40, 41]. These guidelines state that ED is considered a warning sign for early diagnostic or therapeutic intervention. Compared with older antihypertensive drugs, newer agents (ARBs, ACE inhibitors, calcium antagonists, and vasodilating beta-receptor blockers) have neutral or even beneficial effects on ED. Further, the PDE-5 inhibitors offer new avenues in the management of sexual dysfunction.

### Male Hypogonadism, Hypertension and Cardiometabolic Risk

Male hypogonadism is characterized by a deficient production of the male sex hormone testosterone (T)*.* Primary hypogonadism is due to testicular failure, whereas secondary hypogonadism is caused by deficient production of gonadotropins. Compensated hypogonadism is characterized by high luteinizing hormone (LH) levels with normal T.

A clear and internationally recognized definition of male hypogonadism is lacking. Biochemically, T levels below the lower reference of a laboratory cutoff can be considered hypogonadal. Due to significant diurnal variation in T levels, the patient should be fasting, and the blood sample needs to be taken before 10 AM.

The clinical definition of hypogonadism, when considering androgen replacement therapy, is based on both the above biochemical criteria and on the presence of symptoms that can be related to T deficiency.

#### Prevalence of Male Hypogonadism

In young males, hypogonadism is rare, most often related to other comorbidities. The most frequent chromosomal abnormality, Klinefelterʼs syndrome, occurring in approximately 1:600 boys, is frequently associated with primary (testicular) hypogonadism. An increasing proportion of young hypogonadal men are cancer survivors, either those treated for cancer in childhood or testicular cancer, 30–40% of whom have low T levels.

Among adult males, the most frequent cause of hypogonadism is the age-related decline in T levels. In the European Male Aging Study (EMAS), which included more than 3000 men aged 40–80 years recruited from the general population of eight European countries, almost 23% of the participants presented with primary (2%), secondary (9%), or compensated (12%) hypogonadism [42].

The prevalence of primary and compensated, but not secondary, hypogonadism increases with age [43]. Apart from age, comorbidities and high body mass index (BMI) are important risk factors for hypogonadism.

Restricting the definition of hypogonadism to men presenting with both low T and at least three sexual symptoms, the overall prevalence of late-onset hypogonadism in the EMAS study population was 2.1%, increasing from 0.1% for men 40 to 49 years of age to 5.1% for those aged 70 to 79 years [44].

Some studies have indicated a birth-cohort–related negative secular trend in T levels, and it has been suggested that the possible time-related decrease in sperm production is accompanied by increasing Leydig cell functional impairment related to adverse effects of environment and/or lifestyle on the fetal testes [45].

#### Male Hypogonadism and Cardiovascular Risk

Several studies have shown that low T is a marker of decreased life expectancy. During a 25-year follow-up period, men less than 50 years of age with T levels below the 5th percentile experienced twice as high mortality as those with normal hormone levels, and CVD was the most significant single cause of the increased mortality rate [46].

Other studies have shown an association between male childlessness or infertility and risk of CVD, hypertension, and metabolic syndrome [47, 48]. The prevalence of hypogonadism is significantly increased among men with reduced fertility [49]. However, the unresolved question is whether T deficiency is the cause or the consequence of these conditions. It seems likely that hypogonadism may cause CV morbidity since T has been found to have an anti-inflammatory effect and to increase insulin sensitivity [50]. Therefore, a bidirectional association might be plausible, but it cannot be excluded that adverse early life events may play an etiological role for both reproductive dysfunction and CVD risk.

#### Testosterone Replacement in Male Hypogonadism

Testosterone replacement in hypogonadal men aims to normalize serum T levels and improve the subjective symptoms, but there is lack of randomized double-blind placebo-controlled studies documenting the effects of T replacement on comorbidities. The existing data are conflicting, ranging from reports indicating that T is protective against CVD [51] to studies showing the opposite effect [52]. Based on the present level of knowledge, male hypogonadism is considered a marker of increased risk of other diseases, most importantly CVD and the metabolic syndrome. The recommendation is, therefore, that treatment and prevention of metabolic and cardiovascular disorders should be carried out according to current guidelines also in hypogonadic men.

Further, male infertility, as presented by impaired semen quality [53], seeking for infertility treatment [54, 55], or childlessness in married men has been linked to increased risk of cardiometabolic disease and mortality. It is not clear to what extent this association is related to increased prevalence of hypogonadism among men with fertility problems in general, as also other causes may exist [49].

### Female Reproductive Health and Cardiovascular Risk

#### Polycystic Ovary Syndrome

Over the past 25 years, three sets of diagnostic criteria for polycystic ovarian syndrome (PCOS) have been developed by different academic societies and conferences [56]. Using metabolic dysfunction as a remote complication of PCOS, the guidelines published in 2006 recommended that PCOS be defined, first and foremost, as a diagnosis of androgen excess accompanied by either oligo- or anovulation and/or polycystic ovarian morphology after exclusion of related mimicking disorders [57]. The prevalence of PCOS in premenopausal women varies between 4 and 21%, depending on the definition used and the population assessed (referral-based clinical patients or unselected population) [58], possibly making this syndrome the most common endocrine metabolic disorder in women of reproductive age. There is a higher prevalence of overweight and obesity in PCOS women compared with controls [59].

Obese women with PCOS may have higher rates of insulin resistance, hyperinsulinemia, metabolic dysfunction, and hyperandrogenism. There are multiple short-term and long-term consequences of PCOS. The short-term ones include dermatological concerns (hirsutism, acne, androgenic alopecia), reproductive dysfunction (anovulatory infertility in 80% of cases), and mood disturbances (depression or anxiety). Long-term complications include metabolic dysfunction (including a higher risk of developing type 2 diabetes, non-alcoholic fatty liver disease), metabolic syndrome, hypertension, and possible vascular complications [60]. The increased risk of hypertension in PCOS is linked to metabolic dysfunction with mechanisms similar to those in hypogonadal men [61].

Women with PCOS have increased risk for hormone-sensitive neoplasias, including endometrial, ovarian, and breast cancer.

#### Hypertension in Reproductive Age

Women of reproductive age (arbitrarily defined as up to the age of 44 years) have a relatively low prevalence of hypertension, 8–9% [62, 63]. About 50% of hypertensive women of reproductive age are treated with antihypertensive drugs, thus requiring preconception counseling. The prevalence of hypertension is higher in black women and increases with BMI in all ethnicities. Young women with hypertension are at increased risk of developing CVD [64].

Women with chronic hypertension are at risk of experiencing a number of serious complications in pregnancy [65]:

- Superimposed preeclampsia (25.9%; 95% CI 21.0–31.5%)
- Cesarean section (41.4%; 95% CI 35.5–47.7%)
- Preterm delivery < 37 weeks’ gestation (28.1%; 95% CI 22.6–34.4%),
- Birth weight < 2500 g (16.9%; 95% CI 13.1–21.5%)
- Neonatal unit admission (20.5%, 95% CI 15.7–26.4%)
- Perinatal death (4.0%; 95% CI 2.9–5.4%)

The risk of developing preeclampsia can be substantially reduced in women with pre-existing hypertension if low-dose (100–150 mg per day) acetylsalicylic acid (ASA) is initiated from 12 weeks´ gestation until weeks 36–37 [66, 67].

ACE inhibitors, ARBs and direct renin inhibitors are strictly contraindicated in pregnancy, and thus should not be prescribed to women of childbearing potential without reliable contraception. Beta-receptor blockers may induce fetal bradycardia, growth retardation and hypoglycemia;. Consequently, if used, their type and dose of beta-receptor blockers in pregnant women should be carefully selected, with atenolol best avoided [66].

#### Hypertension in Pregnancy

Hypertension complicates 5–10% of pregnancies, with rates likely to increase due to increasing prevalence of obesity and increasing age of pregnant women. Hypertension in pregnancy is not a single entity, and we must distinguish between *pre-existing hypertension* (preceding pregnancy or developing between 20 weeks of gestation; usually persisting more than 42 days postpartum) and *gestational hypertension*, a condition specifically related to hypertension, developing after 20 weeks of gestation and usually resolving within 42 days postpartum. Preeclampsia is defined as gestational hypertension with significant proteinuria (> 0.3 g/24 h or an albumin-to-creatinine ratio ≥ 30 mg/mmol) [66].

Non-pharmacological management of hypertension in pregnancy has a limited role, as randomized studies of dietary and lifestyle interventions showed only minimal effects on pregnancy outcome [68]. Dietary counseling during pregnancy resulted only in a slight reduction of BP (0.66 mmHg for systolic BP and 2.76 mmHg for diastolic BP [69]. Regular exercise may be continued with caution. Obese women should be advised to avoid a weight gain of more than 6.8 kg [70].

There is general consensus that severe hypertension in pregnancy (usually defined by obstetricians as BP ≥160/110 mmHg) should be treated, with values ≥170/110 mmHg considered an emergency when hospitalization is indicated [66]. The selection of antihypertensive drugs and route of their administration depend on the expected time of delivery. ACE inhibitors, ARBs and direct renin inhibitors are contraindicated in pregnancy because of their teratogenic effects. Intravenous labetalol, oral methyldopa or oral nifedipine are recommended. Intravenous hydralazine has been associated with higher rates of perinatal adverse effects and thus should no longer be the drug of choice [71]. Alternatively, intravenous (i.v.) urapidil can be used. Sodium nitroprusside should be reserved for extreme emergencies and administered for the shortest possible period of time because prolonged treatment is associated with increased risk of fetal cyanide poisoning and increased intracranial pressure in the pregnant woman (with potential worsening of cerebral edema). When preeclampsia is associated with pulmonary edema, i.v. nitroglycerine is recommended. Despite lack of evidence, the European guidelines [66, 72] recommend initiation of drug treatment in all women with persistent BP elevations ≥150/95 mmHg and at values >140/90 mmHg in women with:

- Gestational hypertension, or
- Pre-existing hypertension with superimposed gestational hypertension or
- Hypertension with subclinical organ damage or symptoms at any time during pregnancy.

The issue of treatment benefit, tight versus less tight control of hypertension in pregnancy was addressed by the Control of Hypertension in Pregnancy Study (CHIPS). Although the tight control of hypertension was associated with less development of severe maternal hypertension, no difference in the risk of adverse perinatal outcomes and overall serious maternal complications was found [73]. A secondary analysis of data from CHIPS in severe hypertension clearly showed that women developing severe hypertension have higher rates of pregnancy loss or higher neonatal care for longer than 48 h, birth rate less than the 10th percentile, preeclampsia, preterm delivery, platelets less than 10^9^/L, elevated liver enzymes with symptoms, and maternal length of hospital stay of 10 days or longer. Maternal death or serious maternal complications were more common in women with severe hypertension and less tight control [74]. There was also a trend for increased rates of SGA newborns among women in the tight arm who had chronic hypertension [73]. Thus, treatment of maternal hypertension may possibly lead to intrauterine programming, increasing the neonate’s lifetime risk of CVD.

Methyldopa, beta-blockers (most data available for labetalol, a combined alpha- and beta-adrenoreceptor-blocking agent with a more potent effect at beta-receptors in man) and calcium antagonists are the preferred drugs of choice in these guidelines. Magnesium sulfate i.v. is recommended for the prevention of preeclampsia and treatment of seizures (caution if given concomitantly with calcium-channel blockers – risk of hypotension). Women developing gestational hypertension, or preeclampsia in particular, are at increased risk of hypertension, stroke, ischemic heart disease and venous thromboembolism in later life [75–77]. A systematic review and meta-analysis of 22 studies including more than 250,000 women with preeclampsia found preeclampsia to be associated with a fourfold increase in incident heart failure and a twofold increase in the risk of coronary heart disease, stroke and cardiovascular death [78]. Thus, pregnancy offers a unique window for the identification of women at risk of future development of CVD. It is of utmost importance that women with hypertensive disorders in pregnancy are informed about their future risk of developing CVD. There is a need to establish systematic follow-up aimed at detection and control of all major CVD risk factors [79, 80].

#### Assisted Reproductive Technologies (ART)

Children born by these techniques now make up 2–8% of births in developed countries. It is estimated that more than 6 million individuals have been conceived using ART worldwide [81]. Earlier studies did not show an apparent increase in hypertension risk among infertile women or among those with previous fertility treatment [82]. More recently, women who conceived through ART have been shown to be at increased risk of hypertensive disorders in pregnancy. A large retrospective stratified analysis of ART (596,520 mothers; 30.6% ART mothers) indicated that multiple pregnancies after ART are the single most likely explanation for the increased rate of gestational hypertension or preeclampsia among ART mothers [83]. More recently, a meta-analysis including 66 longitudinal studies (7,038,029 pregnancies; 203,375 following any ART) found that all pregnancy-related hypertensive disorders, independent of gestation order, were increased following any invasive ART [+54% (95% CI: 39%–70%), gestational hypertension +79% (95% CI: 24%–157%), and preeclampsia +75% (95% CI: 50%–103%)] [84].

There is growing evidence that ART alters the cardiovascular and metabolic phenotype of offspring [85]. In a recently published Swiss study, 54 young apparently healthy individuals conceived using ART were examined after 5-year follow-up (mean age, 16.5 ± 2.3 years) and compared with age- and sex-matched controls [86]. Flow-mediated dilatation was significantly lower, whereas pulse wave velocity (PWV) and intima-media thickness (IMT) were higher in ART offspring compared with the control group. The significant differences in BP were confirmed by 24-h ambulatory BP monitoring [86].

#### Premature Ovarian Insufficiency

Premature ovarian insufficiency (POI) is a clinical syndrome defined by loss of ovarian activity before the age of 40 years. It is characterized by menstrual disturbances (amenorrhea or oligomenorrhea) with raised gonadotropin and low estradiol levels. The European Society of Human Reproduction and Embryology guideline recommends the following diagnostic criteria: (i) oligo/amenorrhea for at least 4 months and (ii) an elevated follicle-stimulating hormone (FSH) > 25 IU/l on two occasions at least 4 weeks apart [87]. Despite this, approx. 5–10% of women with confirmed POI become pregnant without medical intervention. The estimated prevalence of POI is about 1% in the general population of women in their 40’s. Chromosomal analysis should be performed in all women with non-iatrogenic POI; however, the cause of POI remains not identified in almost 50% of women with POI (idiopathic POI). Women with POI have reduced life expectancy, mostly due to CVD. When diagnosed with POI, the patients should be screened for CVD risk factors on an annual basis with the intent to reduce risk by lifestyle changes. Despite a lack of longitudinal outcome data, early initiated hormone replacement therapy (HRT) is strongly recommended to reduce the risk of developing CVD [87]. Hormone replacement therapy should continue at least until the average age of natural menopause (age 50–51 years in Europe). Patients with Turner syndrome, a genetically determined form of POI, with the most prevalent karyotype 45 (X, 0), have a higher prevalence of aortic coarctation and bicuspid aortic valve, thus increasing the risk of infective endocarditis. Their risk of developing CVD is doubled, compared with individuals with other causes of POI. The typical age of aortic dissection in Turner syndrome is 35 years [88]. Pregnancy imposes stress on the aorta; pregnancy-induced hypertension such as preeclampsia may promote direct vascular damage, aneurysm formation, and, if uncontrolled, induce aortic rupture. Pregnancies in women with Turner syndrome are at a very high risk of both obstetric and non-obstetric complications and should be managed in specialized centers with cardiologist supervision.

#### Oral Contraception and Blood Pressure

The use of combined oral contraceptives is associated with a mild elevation of BP in most women, and overt hypertension may develop in about 5% [89, 90]. Newer preparations with low estrogen and progestogen content seem to be safer. The progestogen-only pill has no effect on BP and is a reasonable alternative for women with hypertension [91]. In a meta-analysis including 24 studies with 270,284 participants, the duration of oral contraceptive use was positively associated with the risk of developing hypertension, increasing by 13% for every 5 years of oral contraceptive use [92].

Blood pressure should be measured at least every 6 months in women using oral contraception. Should BP rise significantly, oral contraceptives should be withdrawn and another contraception technique used [93]. If BP fails to normalize within 3 months of oral contraception withdrawal, the woman should be evaluated and likely treated for hypertension. Oral combined contraception is not recommended for smokers aged over 35 years, for those with systemic autoimmune disease such as systemic lupus erythematosus, or in women with a history of thromboembolic disease. Progestin-only pills, the contraceptive etonogestrel implants or levonorgestrel-releasing intrauterine devices (LNG IUD) might be acceptable alternative options [94]. Oral contraceptives should be given with caution to women with migraine-type attacks [95].

#### BP in the Perimenopausal and Postmenopausal Periods

Hypertension is less prevalent in young premenopausal women than in their male counterparts, there is a steeper increase in systolic BP in women aged 50 to 55 years, resulting in increased prevalence of hypertension in the postmenopausal years. Genetic factors, environmental factors, and change in sex hormone levels contribute to the development of hypertension in postmenopausal women [96]. There is not only a decrease in estradiol and an increase in testosterone but also a change in the estrogen/androgen ratio, resulting in a relative androgen excess that has been proposed to be associated with increased prevalence of hypertension in postmenopausal women [97]. Sex hormone changes are linked with endothelial dysfunction [98], which may also contribute to the development of hypertension after menopause. Endothelial dysfunction is associated with a reduction in NO and an increase in endothelin, both contributing to salt sensitivity. An increase in angiotensin II and endothelin and a reduction in NO may induce increased oxidative stress, thus contributing to an increase in renal vasoconstriction and causing hypertension. Changes in sex hormones are also usually associated with an increase in body weight and sympathetic activation. Further, aging has been found to be associated with a greater increase in sympathetic nervous activity in women than in men [99]. Gender differences in the pathophysiology of hypertension have no major implications for treatment except in the setting of pregnancy. The response to antihypertensive agents and beneficial effects of BP lowering appear to be similar in women and men. However, ACE inhibitors and angiotensin-receptor blockers should be avoided in pregnant women and those of childbearing potential because of their teratogenic effects [100].

In conclusion, hypogonadism is a major determinant of metabolic changes (insulin resistance) and increased prevalence of hypertension both in men (hypoandrogenicity) and women (relative hyperandrogenicity), particularly in the postmenopausal period. The relationship between androgen signaling and NF-kB could possibly explain the pathophysiological mechanisms leading to development of endothelial dysfunction and hypertension [101].

## Conclusions

Human reproduction is of fundamental importance for the survival of mankind. It has been shaped by evolution and resembles that of other mammals in many ways, but not entirely. The role of covert human ovulation (influencing male-female interaction and mate choice) and prolonged breast feeding in women, together with the fact that women live up to 1/3 of their lives in postfertility period, is unique for human beings. Human reproduction is also influenced by societal, cultural, and environmental factors.

The importance of early life programming for risk of developing hypertension and CVD in adults is an impetus for preventive measures to secure maternal and child health—an investment into improved cardiovascular health, in both mothers and their offspring throughout their lives. Efforts to prevent preeclampsia and gestational diabetes are needed, as well as efforts to improve the treatment of these conditions. The discovery of new biomarkers may improve early diagnosis of preeclampsia [102].

A better understanding of these associations could enhance mapping of the mechanisms of BP elevation and vascular aging as well as those promoting cardiometabolic disease. New avenues of research should be promoted, such as the role of gut microbiota in the association between maternal factors and child health [103] as well as increased arterial stiffness in women, as recently reported [104]. Novel treatment for sexual dysfunction, new contraceptive agents, assisted reproduction [105], and strategies for preventing pregnancy complications should be tested in randomized clinical trials, with the ultimate aim to improve reproductive health.

## Abbreviations

ACE: Angiotensin converting enzyme
ARB: Angiotensin receptor blockers
ART: Assisted reproductive technologies
ASA: Acetylsalicylic acid
BMI: Body mass index
BP: Blood pressure
CI: Confidence interval
CVD: Cardiovascular disease
DOHaD: Developmental Origin of Health and Disease
ED: Erectile dysfunction
EMAS: European Male Aging Study
IMT: Intima-media thickness
LH: Luteinizing hormone
LNG IUD: Levonorgestrel-releasing intrauterine devices
ONTARGET: Ongoing Telmisartan Alone and in Combination with Ramipril Global Endpoint Trial
PCOS: Polycystic ovarian syndrome
PDE-5: Phosphodiesterase-5
POI: Premature ovarian insufficiency
PWV: Pulse wave velocity
RAS: Renin-angiotensin system
SGA: Small-for-gestational age
T: Testosterone
TRANSCEND: Telmisartan Randomized Assessment Study in ACE Intolerant Subjects with Cardiovascular Disease
WHO: World Health Organization.

## References

[1] Bolund E, Lummaa V (2017). The effects of resource availability and the demographic transition on the genetic correlation between number of children and grandchildren in humans. Heredity (Edinb).

[2] Courtiol A, Pettay JE, Jokela M, Rotkirch A, Lummaa V (2012). Natural and sexual selection in a monogamous historical human population. Proc Natl Acad Sci U S A.

[3] WHO (2011). Guidelines on preventing early pregnancy and poor reproductive health outcomes among adolescents in developing countries.

[4] Stephenson J, Heslehurst N, Hall J, Schoenaker DAJM, Hutchinson J, Cade JE, Poston L, Barrett G, Crozier SR, Barker M, Kumaran K, Yajnik CS, Baird J, Mishra GD (2018). Before the beginning: nutrition and lifestyle in the preconception period and its importance for future health. Lancet.

[5] Heida KY, Bots ML, de Groot CJ, van Dunné FM, Hammoud NM, Hoek A, Laven JS, Maas AH, Roeters van Lennep J, Velthuis BK, Franx A (2016). Cardiovascular risk management after reproductive and pregnancy-related disorders: a Dutch multidisciplinary evidence-based guideline. Eur J Prev Cardiol.

[6] Muijsers HEC, Roeleveld N, van der Heijden OWH (2019). Consider preeclampsia as a first cardiovascular event. Curr Cardiovasc Risk Rep.

[7] Gandaglia G, Briganti A, Jackson G, Kloner RA, Montorsi F, Montorsi P, Vlachopoulos C (2014). A systematic review of the association between erectile dysfunction and cardiovascular disease. Eur Urol.

[8] Capogrosso P, Ventimiglia E, Boeri L, Cazzaniga W, Chierigo F, Montorsi F, Salonia A (2018). Male infertility as a proxy of the overall male health status. Minerva Urol Nefrol.

[9] Gennser G, Rymark P, Isberg PE (1988). Low birth weight and risk of high blood pressure in adulthood. Br Med J (Clin Res Ed).

[10] Barker DJ, Bull AR, Osmond C, Simmonds SJ (1990). Fetal and placental size and risk of hypertension in adult life. BMJ..

[11] Koupilová I, Leon DA, Lithell HO, Berglund L (1997). Size at birth and hypertension in longitudinally followed 50-70-year-old men. Blood Press.

[12] Nilsson PM, Ostergren PO, Nyberg P, Söderström M, Allebeck P (1997). Low birth weight is associated with elevated systolic blood pressure in adolescence: a prospective study of a birth cohort of 149378 Swedish boys. J Hypertens.

[13] Law CM, Shiell AW (1996). Is blood pressure inversely related to birth weight? The strength of evidence from a systematic review of the literature. J Hypertens.

[14] Huxley RR, Shiell AW, Law CM (2000). The role of size at birth and postnatal catch-up growth in determining systolic blood pressure: a systematic review of the literature. J Hypertens.

[15] Libby G, McEwan SR, Belch JJ, Morris AD (2008). Birth weight does not predict blood pressure in a young working population: a sharp (Scottish Heart and Arterial Disease Risk Prevention) study. Ann Epidemiol.

[16] Winder NR, Krishnaveni GV, Hill JC, Karat CL, Fall CH, Veena SR, Barker DJ (2011). Placental programming of blood pressure in Indian children. Acta Paediatr.

[17] Hult M, Tornhammar P, Ueda P, Chima C, Bonamy AK, Ozumba B, Norman M (2010). Hypertension, diabetes and overweight: looming legacies of the Biafran famine. PLoS One.

[18] Barker DJ, Osmond C, Law CM (1989). The intrauterine and early postnatal origins of cardiovascular disease and chronic bronchitis. J Epidemiol Community Health.

[19] Phipps K, Barker DJ, Hales CN, Fall CH, Osmond C, Clark PM (1993). Fetal growth and impaired glucose tolerance in men and women. Diabetologia.

[20] Gluckman PD, Hanson MA, Cooper C, Thornburg KL (2008). Effect of in utero and early-life conditions on adult health and disease. N Engl J Med.

[21] Phillips DI, Walker BR, Reynolds RM, Flanagan DE, Wood PJ, Osmond C, Barker DJ, Whorwood CB (2000). Low birth weight predicts elevated plasma cortisol concentrations in adults from 3 populations. Hypertension.

[22] Baum M (2018). Role of renal sympathetic nerve activity in prenatal programming of hypertension. Pediatr Nephrol.

[23] Zohdi V, Sutherland MR, Lim K, Gubhaju L, Zimanyi MA, Black MJ (2012). Low birth weight due to intrauterine growth restriction and/or preterm birth: effects on nephron number and long-term renal health. Int J Nephrol.

[24] McKeigue PM, Lithell HO, Leon DA (1998). Glucose tolerance and resistance to insulin-stimulated glucose uptake in men aged 70 years in relation to size at birth. Diabetologia..

[25] Warrington NM, Beaumont RN, Horikoshi M, Day FR, Helgeland Ø, Laurin C, Bacelis J, Peng S, Hao K, Feenstra B, Wood AR, Mahajan A, Tyrrell J, Robertson NR, Rayner NW, Qiao Z, Moen GH, Vaudel M, Marsit CJ, Chen J, Nodzenski M, Schnurr TM, Zafarmand MH, Bradfield JP, Grarup N, Kooijman MN, Li-Gao R, Geller F, Ahluwalia TS, Paternoster L, Rueedi R, Huikari V, Hottenga JJ, Lyytikäinen LP, Cavadino A, Metrustry S, Cousminer DL, Wu Y, Thiering E, Wang CA, Have CT, Vilor-Tejedor N, Joshi PK, Painter JN, Ntalla I, Myhre R, Pitkänen N, van Leeuwen E, Joro R, Lagou V, Richmond RC, Espinosa A, Barton SJ, Inskip HM, Holloway JW, Santa-Marina L, Estivill X, Ang W, Marsh JA, Reichetzeder C, Marullo L, Hocher B, Lunetta KL, Murabito JM, Relton CL, Kogevinas M, Chatzi L, Allard C, Bouchard L, Hivert MF, Zhang G, Muglia LJ, Heikkinen J, Morgen CS, van Kampen A, van Schaik B, Mentch FD, Langenberg C, Luan J, Scott RA, Zhao JH, Hemani G, Ring SM, Bennett AJ, Gaulton KJ, Fernandez-Tajes J, van Zuydam N, Medina-Gomez C, de Haan HG, Rosendaal FR, Kutalik Z, Marques-Vidal P, Das S, Willemsen G, Mbarek H, Müller-Nurasyid M, Standl M, Appel EVR, Fonvig CE, Trier C, van Beijsterveldt C, Murcia M, Bustamante M, Bonas-Guarch S, Hougaard DM, Mercader JM, Linneberg A, Schraut KE, Lind PA, Medland SE, Shields BM, Knight BA, Chai JF, Panoutsopoulou K, Bartels M, Sánchez F, Stokholm J, Torrents D, Vinding RK, Willems SM, Atalay M, Chawes BL, Kovacs P, Prokopenko I, Tuke MA, Yaghootkar H, Ruth KS, Jones SE, Loh PR, Murray A, Weedon MN, Tönjes A, Stumvoll M, Michaelsen KF, Eloranta AM, Lakka TA, van Duijn C, Kiess W, Körner A, Niinikoski H, Pahkala K, Raitakari OT, Jacobsson B, Zeggini E, Dedoussis GV, Teo YY, Saw SM, Montgomery GW, Campbell H, Wilson JF, Vrijkotte TGM, Vrijheid M, de Geus EJCN, Hayes MG, Kadarmideen HN, Holm JC, Beilin LJ, Pennell CE, Heinrich J, Adair LS, Borja JB, Mohlke KL, Eriksson JG, Widén EE, Hattersley AT, Spector TD, Kähönen M, Viikari JS, Lehtimäki T, Boomsma DI, Sebert S, Vollenweider P, Sørensen TIA, Bisgaard H, Bønnelykke K, Murray JC, Melbye M, Nohr EA, Mook-Kanamori DO, Rivadeneira F, Hofman A, Felix JF, Jaddoe VWV, Hansen T, Pisinger C, Vaag AA, Pedersen O, Uitterlinden AG, Järvelin MR, Power C, Hyppönen E, Scholtens DM, Lowe WL Jr, Davey Smith G, Timpson NJ, Morris AP, Wareham NJ, Hakonarson H, Grant SFA, Frayling TM, Lawlor DA, Njølstad PR, Johansson S, Ong KK, McCarthy M, Perry JRB, Evans DM, Freathy RM, EGG Consortium (2019). Maternal and fetal genetic effects on birth weight and their relevance to cardio-metabolic risk factors. Nat Genet.

[26] Nilsson PM, Lurbe E, Laurent S (2008). The early life origins of vascular ageing and cardiovascular risk: the EVA syndrome. J Hypertens.

[27] Sperling J, Nilsson PM. Does early life programming influence arterial stiffness and central hemodynamics in adulthood? J Hypertens. 2020;38(3):481–8. 10.1097/HJH.0000000000002292 31633583

[28] Feldman HA, Goldstein I, Hatzichristou DG, Krane RJ, McKinlay JB (1994). Impotence and its medical and psychosocial correlates: results of the Massachusetts male aging study. J Urol.

[29] Viigimaa M, Vlachopoulos C, Lazaridis A, Doumas M (2014). Management of erectile dysfunction in hypertension: tips and tricks. World J Cardiol.

[30] Mancia G, Fagard R, Narkiewicz K, Redon J, Zanchetti A, Böhm M (2013). 2013 ESH/ESC Guidelines for the management of arterial hypertension: the task force for the management of arterial hypertension of the European Society of Hypertension (ESH) and of the European Society of Cardiology (ESC). J Hypertens.

[31] Manolis A, Doumas M (2012). Antihypertensive treatment and sexual dysfunction. Curr Hypertens Rep.

[32] Montorsi P, Ravagnani PM, Galli S, Rotatori F, Briganti A, Salonia A, Rigatti P, Montorsi F (2005). The artery size hypothesis: a macrovascular link between erectile dysfunction and coronary artery disease. Am J Cardiol.

[33] Gupta BP, Murad MH, Clifton MM, Prokop L, Nehra A, Kopecky SL (2011). The effect of lifestyle modification and cardiovascular risk factor reduction on erectile dysfunction: a systematic review and meta-analysis. Arch Intern Med.

[34] Viigimaa M, Doumas M, Vlachopoulos C, Anyfanti P, Wolf J, Narkiewicz K (2011). Hypertension and sexual dysfunction: time to act. J Hypertens.

[35] Doumas M, Viigimaa M, Papademetriou V (2013). Combined antihypertensive therapy and sexual dysfunction: terra incognita. Cardiology..

[36] Grimm RH, Grandits GA, Prineas RJ, McDonald RH, Lewis CE, Flack JM (1997). Long-term effects on sexual function of five antihypertensive drugs and nutritional hygienic treatment in hypertensive men and women. Treatment of Mild Hypertension Study (TOMHS). Hypertension.

[37] Dusing R (2003). Effect of the angiotensin II antagonist valsartan on sexual function in hypertensive men. Blood Pressure (Suppl).

[38] Böhm M, Baumhäkel M, Teo K, Sleight P, Probstfield J, Gao P (2010). Erectile dysfunction predicts cardiovascular events in high-risk patients receiving telmisartan, ramipril, or both: the Ongoing Telmisartan Alone and in combination with Ramipril Global Endpoint Trial/Telmisartan Randomized AssessmeNT Study in ACE iNtolerant subjects with cardiovascular Disease (ONTARGET/TRANCEND) trials. Circulation.

[39] Scranton RE, Lawler E, Botteman M, Chittamooru S, Gagnon D, Lew R, Harnett J, Gaziano JM (2007). Effect of treating erectile dysfunction on management of systolic hypertension. Am J Cardiol.

[40] Piepoli MF, Hoes AW, Agewall S, Albus C, Brotons C, Catapano AL (2016). European Guidelines on cardiovascular disease prevention in clinical practice. Eur Heart J.

[41] Whelton PK, Carey RM, Aronow WS, Casey DE, Collins KJ (2017). Guideline for the prevention, detection, evaluation, and management of high blood pressure in adults: a report of the American College of Cardiology/American Heart Association Task Force on Clinical Practice Guidelines. J Am Coll Cardiol.

[42] Wu FC, Tajar A, Beynon JM (2010). Identification of late-onset hypogonadism in middle-aged and elderly men. N Engl J Med.

[43] Wu FC, Tajar A, Pye SR, Silman AJ, Finn JD, O’Neill TW, Bartfai G, Casanueva F, Forti G, Giwercman A, Huhtaniemi IT, Kula K, Punab M, Boonen S, Vanderschueren D, European Male Aging Study Group (2008). Hypothalamic-pituitary-testicular axis disruptions in older men are differentially linked to age and modifiable risk factors: the European Male Aging Study. J Clin Endocrinol Metab.

[44] Tajar A, Forti G, O'Neill TW, Lee DM, Silman AJ, Finn JD, Bartfai G, Boonen S, Casanueva FF, Giwercman A, Han TS, Kula K, Labrie F, Lean ME, Pendleton N, Punab M, Vanderschueren D, Huhtaniemi IT, Wu FC, EMAS Group (2010). Characteristics of secondary, primary, and compensated hypogonadism in aging men: evidence from the European Male Ageing Study. J Clin Endocrinol Metab.

[45] Andersson AM, Jensen TK, Juul A, Petersen JH, Jorgensen T, Skakkebaek NE (2007). Secular decline in male testosterone and sex hormone binding globulin serum levels in Danish population surveys. J Clin Endocrinol Metab.

[46] Bentmar Holgersson M, Landgren F, Rylander L, Lundberg Giwercman Y (2017). Mortality is linked to low serum testosterone levels in younger and middle-aged men. Eur Urol.

[47] Bungum AB, Glazer CH, Bonde JP, Nilsson PM, Giwercman A, Sogaard Tottenborg S (2018). Risk of metabolic disorders in childless men: a population-based cohort study. BMJ Open.

[48] Elenkov A, Al-Jebari Y, Giwercman A (2018). More prevalent prescription of medicine for hypertension and metabolic syndrome in males from couples undergoing Intracytoplasmic sperm injection. Sci Rep.

[49] Bobjer J, Bogefors K, Isaksson S, Leijonhufvud I, Akesson K, Giwercman YL (2016). High prevalence of hypogonadism and associated impaired metabolic and bone mineral status in subfertile men. Clin Endocrinol.

[50] Bobjer J, Katrinaki M, Tsatsanis C, Lundberg Giwercman Y, Giwercman A (2013). Negative association between testosterone concentration and inflammatory markers in young men: a nested cross-sectional study. PLoS One.

[51] Jones TH, Kelly DM (2018). Randomized controlled trials - mechanistic studies of testosterone and the cardiovascular system. Asian J Androl.

[52] Basaria S, Coviello AD, Travison TG (2010). Adverse events associated with testosterone administration. N Engl J Med.

[53] Jensen TK, Jacobsen R, Christensen K, Nielsen NC, Bostofte E (2009). Good semen quality and life expectancy: a cohort study of 43,277 men. Am J Epidemiol.

[54] Glazer CH, Bonde JP, Eisenberg ML, Giwercman A, Haervig KK, Rimborg S (2017). Male infertility and risk of nonmalignant chronic diseases: a systematic review of the epidemiological evidence. Semin Reprod Med.

[55] Glazer CH, Bonde JP, Giwercman A, Vassard D, Pinborg A, Schmidt L, Vaclavik Brauner E (2017). Risk of diabetes according to male factor infertility: a register-based cohort study. Hum Reprod.

[56] Escobar-Morreale HF (2018). Polycystic ovary syndrome: definition, aetiology, diagnosis and treatment. Nat Rev Endocrinol.

[57] Azziz R, Carmina E, Dewailly D, Diamanti-Kandarakis E, Escobar-Morreale HF, Futterweit W (2006). Positions statement: criteria for defining polycystic ovary syndrome as a predominantly hyperandrogenic syndrome: an androgen excess society guideline. J Clin Endocrinol Metab.

[58] Sirmans SM, Pate KA (2013). Epidemiology, diagnosis, and management of polycystic ovary syndrome. Clin Epidemiol.

[59] Carmina E, Campagna AM, Lobo RA (2012). A 20-year follow-up of young women with polycystic ovary syndrome. Obstet Gynecol.

[60] Azziz R, Carmina E, Chen Z, Dunaif A, Laven JS, Legro RS (2016). Polycystic ovary syndrome. Nat Rev Dis Primers.

[61] Moulana M, Lima R, Reckelhoff JF (2011). Metabolic syndrome, androgens, and hypertension. Curr Hypertens Rep.

[62] Bateman BT, Shaw KM, Kuklina EV, Callaghan WM, Seely EW, Hernández-Díaz S (2012). Hypertension in women of reproductive age in the United States: NHANES 1999-2008. PLoS One.

[63] Azeez O, Kulkarni A, Kuklina EV, Kim SY, Cox S (2019). Hypertension and diabetes in non-pregnant women of reproductive age in the United States. Prev Chronic Dis.

[64] Ford ES, Capewell S (2007). Coronary heart disease mortality among young adults in the U.S. from 1980 through 2002: concealed leveling of mortality rates. J Am Coll Cardiol.

[65] Bramham K, Parnell B, Nelson-Piercy C, Seed PT, Poston L, Chappell LC (2014). Chronic hypertension and pregnancy outcomes: systematic review and meta-analysis. BMJ.

[66] Regitz-Zagrosek V, Roos-Hesselink JW, Bauersachs J, Blomström-Lundqvist C, Cífková R, De Bonis M (2018). 2018 ESC guidelines for the management of cardiovascular diseases during pregnancy. Eur Heart J.

[67] Rolnik DL, Wright D, Poon LC, O’Gorman N, Syngelaki A, de Paco Matallana C (2017). Aspirin versus placebo in pregnancies at high-risk for preterm preeclampsia. N Engl J Med.

[68] Dodd JM, Turnbull D, McPhee AJ, Deussen AR, Grivell RM, Yelland LN (2014). Antenatal lifestyle advice for women who are overweight or obese: LIMIT randomised trial. BMJ.

[69] Gresham E, Bisquera A, Byles JE, Hure AJ (2016). Effects of dietary interventions on pregnancy outcomes: a systematic review and meta-analysis. Matern Child Nutr.

[70] Leddy MA, Power ML, Schulkin J (2008). The impact of maternal obesity on maternal and fetal health. Rev Obstet Gynecol.

[71] Magee LA, Cham C, Waterman EJ, Ohlsson A, von Dadelszen P (2003). Hydralazine for treatment of severe hypertension in pregnancy: meta-analysis. BMJ.

[72] Williams B, Mancia G, Spiering W, Agabiti Rosei E, Azizi M, Burnier M (2018). 2018 ESC/ESH Guidelines for the management of arterial hypertension: the task force for the management of arterial hypertension of the European Society of Cardiology and the European Society of Hypertension: the task force for the management of arterial hypertension of the European Society of Cardiology and the European Society of Hypertension. J Hypertens.

[73] Magee LA, von Dadelszen P, Rey E, Ross S, Asztalos E, Murphy KE, Menzies J, Sanchez J, Singer J, Gafni A, Gruslin A, Helewa M, Hutton E, Lee SK, Lee T, Logan AG, Ganzevoort W, Welch R, Thornton JG, Moutquin JM (2015). Less-tight versus tight control of hypertension in pregnancy. N Engl J Med.

[74] Magee LA, von Dadelszen P, Singer J, Lee T, Rey E, Ross S (2016). The CHIPS randomized controlled trial (control of hypertension in pregnancy study): is severe hypertension just an elevated blood pressure?. Hypertension.

[75] Bellamy L, Casas JP, Hingorani AD, Williams DJ (2007). Pre-eclampsia and risk of cardiovascular disease and cancer in later life: systematic review and meta-analysis. BMJ..

[76] Ray JG, Vermeulen MJ, Schull MJ, Redelmeier DA (2005). Cardiovascular health after maternal placental syndromes (CHAMPS): population-based retrospective cohort study. Lancet.

[77] Black MH, Zhou H, Sacks DA, Dublin S, Lawrence JM, Harrison TN, Reynolds K (2016). Hypertensive disorders first identified in pregnancy increase risk for incident prehypertension and hypertension in the year after delivery. J Hypertens.

[78] Wu P, Haththotuwa R, Kwok CS, Babu A, Kotronias RA, Rushton C (2017). Preeclampsia and future cardiovascular health: a systematic review and meta-analysis. Circ Cardiovasc Qual Outcomes.

[79] Cífková R (2018). Cardiovascular sequels of hypertension in pregnancy. J Am Heart Assoc.

[80] Smith GN, Louis JM, Saade GR (2019). Pregnancy and the postpartum period as an opportunity for cardiovascular risk identification and management. Obstet Gynecol.

[81] Kupka MS, D'Hooghe T, Ferraretti AP, de Mouzon J, Erb K, Castilla JA, European IVF-Monitoring Consortium (EIM); European Society of Human Reproduction and Embryology (ESHRE) (2016). Assisted reproductive technology in Europe, 2011: results generated from European registers by ESHRE. Hum Reprod.

[82] Farland LV, Grodstein F, Srouji SS, Forman JP, Rich-Edwards J, Chavarro JE, Missmer SA (2015). Infertility, fertility treatment, and risk of hypertension. Fertil Steril.

[83] Wang YA, Chughtai AA, Farquhar CM, Pollock W, Lui K, Sullivan EA (2016). Increased incidence of gestational hypertension and preeclampsia after assisted reproductive technology treatment. Fertil Steril.

[84] Thomopoulos C, Salamalekis G, Kintis K, Andrianopoulou I, Michalopoulou H, Skalis G (2017). Risk of hypertensive disorders in pregnancy following assisted reproductive technology: overview and meta-analysis. J Clin Hypertens (Greenwich).

[85] Ceelen M, van Weissenbruch MM, Vermeiden JP, van Leeuwen FE, Delemarre-van de Waal HA (2008). Cardiometabolic differences in children born after in vitro fertilization: follow-up study. J Clin Endocrinol Metab.

[86] Meister TA, Rimoldi SF, Soria R, von Arx R, Messerli FH, Sartori C, Scherrer U, Rexhaj E (2018). Association of Assisted Reproductive Technologies with arterial hypertension during adolescence. J Am Coll Cardiol.

[87] Webber L, Davies M, Anderson R, Bartlett J, Braat D, Cartwright B, European Society for Human Reproduction and Embryology (ESHRE) Guideline Group on POI (2016). ESHRE guideline: management of women with premature ovarian insufficiency. Hum Reprod.

[88] Gravholt CH, Landin-Wilhelmsen K, Stochholm K, Hjerrild BE, Ledet T, Djurhuus CB, Sylvén L, Baandrup U, Kristensen BØ, Christiansen JS (2006). Clinical and epidemiological description of aortic dissection in Turner’s syndrome. Cardiol Young.

[89] Chasan-Taber L, Willett WC, Manson JE, Spiegelman D, Hunter DJ, Curhan G, Colditz GA, Stampfer MJ (1996). Prospective study of oral contraceptives and hypertension among women in the United States. Circulation.

[90] Dong W, Colhoun HM, Poulter NR (1997). Blood pressure in women using oral contraceptives: results from the health survey for England 1994. J Hypertens.

[91] Hussain SF (2004). Progestogen-only pills and high blood pressure: is there an association? A literature review. Contraception.

[92] Liu H, Yao J, Wang W, Zhang D (2017). Association between duration of oral contraceptive use and risk of hypertension: a meta-analysis. J Clin Hypertens (Greenwich).

[93] Lubianca JN, Moreira LB, Gus M, Fuchs FD (2005). Stopping oral contraceptives: an effective blood pressure-lowering intervention in women with hypertension. J Hum Hypertens.

[94] ACOG (2019). Practice Bulletin No. 206: use of hormonal contraception in women with coexisting medical conditions. Obstet Gynecol.

[95] World Health Organization. Medical eligibility criteria for contraceptive use. Third edition, 2004. http://apps.who.int/iris/bitstream/10665/42907/1/9241562668.pdf (date accessed June 28th 2018).

[96] Coylewright M, Reckelhoff JF, Ouyang P (2008). Menopause and hypertension: an age-old debate. Hypertension.

[97] Yanes LL, Reckelhoff JF (2011). Postmenopausal hypertension. Am J Hypertens.

[98] Taddei S, Virdis A, Ghiadoni L, Mattei P, Sudano I, Bernini G, Pinto S, Salvetti A (1996). Menopause is associated with endothelial dysfunction in women. Hypertension.

[99] Narkiewicz K, Phillips BG, Kato M, Hering D, Bieniaszewski L, Somers VK (2005). Gender-selective interaction between aging, blood pressure, and sympathetic nerve activity. Hypertension.

[100] Turnbull F, Woodward M, Neal B, Barzi F, Ninomiya T, Chalmers J, Perkovic V, Li N, MacMahon S, Blood Pressure Lowering Treatment Trialists’ Collaboration; Blood Pressure Lowering Treatment Trialists’ Collaboration (2008). Do men and women respond differently to blood pressure-lowering treatment? Results of prospectively designed overviews of randomized trials. Eur Heart J.

[101] Moretti C, Lanzolla G, Moretti M, Gnessi L, Carmina E (2017). Androgens and hypertension in men and women: a unifying view. Curr Hypertens Rep.

[102] Anderson UD, Gram M, Ranstam J, Thilaganathan B, Kerström B, Hansson SR (2016). Fetal hemoglobin, α1-microglobulin and hemopexin are potential predictive first trimester biomarkers for preeclampsia. Pregnancy Hypertens.

[103] Tun HM, Bridgman SL, Chari R, Field CJ, Guttman DS, Becker AB (2018). Roles of birth mode and infant gut microbiota in intergenerational transmission of overweight and obesity from mother to offspring. JAMA Pediatr.

[104] Menni C, Lin C, Cecelja M, Mangino M, Matey-Hernandez ML, Keehn L, Mohney RP, Steves CJ, Spector TD, Kuo CF, Chowienczyk P, Valdes AM (2018). Gut microbial diversity is associated with lower arterial stiffness in women. Eur Heart J.

[105] García D, Massucci FA, Mosca A, Ràfols I, Rodríguez A, Vassena R. Mapping research in assisted reproduction worldwide. Reprod Biomed Online. 2019;40(1):71–81. 10.1016/j.rbmo.2019.10.013 31862416

